# Evaluation of Intraocular pressure, Corneal thickness, and Retinal nerve fiber layer thickness in patients with Obstructive Sleep Apnea Syndrome

**DOI:** 10.12669/pjms.344.15018

**Published:** 2018

**Authors:** Kuddusi Teberik, Mehmet Tahir Eski, Ege Gulec Balbay, Murat Kaya

**Affiliations:** 1 Kuddusi Teberik, MD.Assistant Professor, Department of Ophthalmology, Duzce University Medical School, Duzce, Turkey; 2 Mehmet Tahir Eski, MD.Ministry of Health Bingol State Hospital, Bingol, Turkey; 3 Ege Gulec Balbay, MD.Associate Professor, Department of Chest Diseases, Duzce University Medical School, Duzce, Turkey; 4 Murat Kaya, MD.Professor, Department of Ophthalmology, Duzce University Medical School, Duzce, Turkey

**Keywords:** Obstructive sleep apnea syndrome, Spectral-domain optical coherence tomography, Central corneal thickness, Retinal nerve fiber layer thickness

## Abstract

**Objective:**

To evaluate the intraocular pressure (IOP), central corneal thickness (CCT), and peripapillary retinal nerve fiber layer (RNFL) thickness in Patients with Obstructive Sleep Apnea Syndrome.

**Methods:**

In this prospective study, 103 patients with OSAS (study group) and 37 healthy subjects were enrolled. All participants underwent comprehensive ophthalmic examinations. Mean outcome measures were intraocular pressure by Goldmann applanation tonometry, CCT measurement using ultrasound pachymeter and peripapillary RNFL thickness measured by spectral-domain optical coherence tomography.

**Results:**

The differences between the mean values of RNFL thickness in all quadrants were similar in both groups and were not statistically significant (p=0.274). The IOP and CCT measurement averages of all patients with OSAS were lower than the control group. However, this difference was not statistically significant. There was no correlation between the apnea-hypopnea index, lowest oxygen saturation (LAST) or Body Mass Index (BMI) and the peripapillary RNFL thickness, IOP or CCT when OSAS group was divided by severity.

**Conclusions:**

The study results suggest that peripapillary RNFL thickness, IOP or CCT did not differ significantly between OSAS and control groups. We also found no correlation between apnea severity (AHI), lowest oxygen saturation (LAST) and BMI and RNFL, CCT and IOP.

## INTRODUCTION

Obstructive Sleep Apnea Syndrome (OSAS) is part of a broad group of disorders known as “sleep-related breathing disorders”. OSAS is described by brief scenes of finished or incomplete upper airway collapse while sleeping, bringing about an expanded thoracoabdominal exertion and a diminished blood vessel oxygen immersion, prompting an excitement reaction which appears as apneas and occasional hypopneas while sleeping.^1^ OSAS is a common sleep disorder that is probable to be seen in genders, ages, socioeconomic statuses and ethnic groups.^2^ OSAS is a disease which affects 24% of middle aged men and 9% of women all around the World, and minimum 2-4% of the adults are under the influence of symptomatic OSAS.^3^,^4^ Epidemiological studies in Turkey show that the percentage of prevalence of OSAS is 1.8%.^5^ The main symptoms are excessive daytime sleepiness, fatigue, and impaired cognitive abilities. Diagnosis of OSAS is made by nighttime polysomnography. Apnea Hypopnea Index (AHI), a widely used indicator of the severity of OSAS, was defined as some hypopnea or apnea per hour. At the point when the AHI is no less than 5 events/h, OSAS is viewed as a clinical substance.^6^ It was reported that OSAS was essentially associated with a few neurodegenerative disorders, for example, Alzheimer’s disease, Parkinson disease.^7^-^9^ Ophthalmological disorders such as non-arteritic anterior ischemic optic neuropathy (NAION), central serous retinopathy, floppy eyelid syndrome, primary open-angle glaucoma, normal tension glaucoma, and papilledema are in the reports of the patients who are suffering from the effects of OSAS.^10^,^11^

Obstructive respiratory disorders increase vascular resistance due to severe hypoxemia. This can lead to optic neuropathy by disturbing the perfusion of the optic nerve head. It is not fully explained between ischemia and glaucoma. Studies have shown, however, that glaucoma-related risk factors are present in patients with OSAS.

In this respect, intraocular pressure (IOP), central corneal thickness (CCT) and peripapillary retinal nerve fiber layer (RNFL) thickness can be altered in this disease. We aimed to determine whether there was a correlation between the clinical severity of OSAS and the measured values of IOP, CCT and peripapillary RNFL thickness in our study.

## METHODS

This prospective, case-control study was performed in the Department of Pulmonary Disease and Ophthalmology Clinics, Medical School of Duzce University (Duzce, Turkey) between January 2017 and June 2017. The study protocol was approved by the local ethics committee, and all participants provided written informed consent according to the Declaration of Helsinki. One hundred and three patients who were newly diagnosed with OSAS comprised in the OSAS group. A control group was formed with 37 healthy individuals.

### Exclusion Criteria

For both study groups, we excluded patients who had a history of ocular surgery, ocular trauma, anterior or posterior segment disease, glaucoma, bronchial asthma, interstitial lung diseases, cerebrovascular disease, systemic hypertension, and diabetes. Patients who were using any drugs and patients with any refractive errors higher than 2.00 Diopters (D) were also excluded. The subjects in the control group had no health problems, but they were not monitored during sleep to time and resources. Any volunteer participant meeting inclusion and exclusion criteria were questioned about their history of daytime sleepiness, snoring, or episodes of breathing cessation during sleep witnessed by another person. Patients who answered yes to any of these questions were not included in the study.

### Ophthalmological Examination

All OSAS patients and control subjects underwent a complete ocular examination, including assessment of best corrected visual acuity (BCVA), ocular motility, pupillary reflexes, slit-lamp biomicroscopy, IOP measurement with Goldmann applanation tonometry, gonioscopy with a three-mirror contact lenses, ultrasound CCT measurement (Echoscan US 500; Nidek Co. Ltd, Aichi, Japan), and dilated fundus examination. Peripapillary RNFL (G:global, T:temporal, Ts:temporal superior, Ti:temporal inferior, N:nasal, Ns:nasal superior, Ni:nasal inferior) measurements were obtained using EDI-OCT (SD-OCT; Heidelberg Engineering, Heidelberg, Germany). Ophthalmological examinations and OCT scans were performed by an experienced ophthalmologist unaware of clinical information of the participants. We performed all measurements three times and used the average of the measurements for the statistical analyses.

### Polysomnography & Sleep Assessment

Full overnight polysomnography (Philips Respironics Model: Alice-6 PSG, German) procedure was performed to patients who were referred to sleep laboratories with a preliminary diagnosis of OSAS. The system uses dual-channel EEG (electroencephalography), 2-channel EOG (electrooculography), submental electromyelography (EMG), tibial EMG, oral and nasal airflow (by the thermistor and nasal cannula), thoracic and abdominal movements, body position, snoring, ECG and pulse oximetry recordings were obtained (> 6 hours). All recordings were manually scored by a certified sleep physician. Apnea was defined as complete cessation of oral and nasal airflow for at least 10 seconds. Hypopnea was defined as a reduction > 30% in airflow for at least 10 seconds accompanied by > 3% desaturation and arousal.

The average number of episodes of apnea and hypopnea per hour of sleep were measured according to the Apnea/Hypopnea Index (AHI). Patients with an AHI <5 were considered OSAS-negative, while those with AHI 5-15 were regarded as having mild, AHI 15-30 to have moderate and AHI >30 to have severe OSAS.

### Statistical Analyses

Statistical analysis was performed using SPSS for Windows version 17 software (SPSS Inc, Chicago, IL, USA). The descriptive statistics for continuous variables were expressed in the number percentage (%) the significance of the difference between the mean values of the groups was evaluated using the Stundent’s t-test, while the significance of the difference in the median values was evaluated using the Mann-Whitney U test. Pearson’s correlation efficient was used to measure strength and direction of the linear relationship between two variables. A p value less than 0.05 was considered statistically significant.

## RESULTS

In all, 103 patients with OSAS (28 mild’s, 31 moderate and 44 severe) and 37 controls were included in the study. The mean age was 50.1 ± 6.9 years in the control group and 53.3 ± 11.9 years in the total OSAS group (p=0.016). The features of the patients and control are summarised in (Table-I).

**Table-I:** Demographic data of OSAS patients and controls.

	Mild OSAS	Moderate OSAS	Severe OSAS	Total OSAS	Controls
Number of the patients	27	32	44	103	37
Age (years)	50.3 ± 13.1	51.2 ± 11.1	56.8 ± 11.0	53.3 ± 11.9	50.1 ± 6.9	
	(27-68)	(35-69)	(37-79)	(27-79)	(37-68)	
**Sex**:					
Male	15 (53.7 %)	21 (67.7 %)	36 (81.8 %)	71 (68.9 %)	28 (75.6 %)
Female	13 (46.4 %)	10 (32.2 %)	8 (18.2 %)	32 (31.1 %)	9 (24.4 %)

OSAS: Obstructive sleep apnea syndrome.

Polysomnographic data of the patients with OSAS subgroups is shown in Table-II. No significant difference was found between mild and moderate OSAS groups for AHI (p=0.035), but there was a statistically significant difference between both mild and severe OSAS groups and between moderate and severe OSAS groups (p < 0.001, P< 0.001, respectively).

**Table-II:** Comparison of polysomnographic data in OSAS patients according to the disease severity.

	Mild OSAS cases	Moderate OSAS cases	Severe OSAS cases	P value
AHI	11.60 ± 2.73	22.1 ± 4.4	61.2 ± 24.4	<0.001^a,b^
	(5-15)	(15.9-29.5)	(30.8-132.3)	
BMI (kg/m^2^)	32.5 ± 9.1	33.1 ± 9.2	36.2 ± 8.1	0.149
	(23.9-58.1)	(24.1-62)	(24.1-61.3)	
lowest oxygen saturation (LAST)	85.7 ± 5.9	79.9 ± 12.2	73.8 ± 15.6	<0.001^a^
	(70-92)	(39-91)	(20-94)	

AHI: Apnea-hypopnea index; BMI: Body mass index;

a According to post hoc analysis, statistically significant difference between the mild and severe groups

b According to post hoc analysis, statistically significant difference between the moderate and severe groups.

No significant difference was found between OSAS stages in terms of body mass index (BMI). There was a statistically significant difference between the mild and severe OSAS groups for the lowest oxygen saturation (LAST) (p<0.001), but no significant difference was found among the others (Table-III).

**Table-III:** Comparison of IOP, CCT, K1, K2, ACD, AL and peripapillary RNFL (G,T,Ts,Ti,N,Ns,Ni) thickness measurements between OSAS patients and controls.

	Mild OSAS	Moderate OSAS	Severe OSAS	Controls
IOP (mmHg)	15.0 ± 2.7	15.1 ± 3.1	15.8 ± 2.1	16.1 ± 1.8
CCT (μm)	539.8 ± 30.2	548.0 ± 32.7	549.2 ± 42.3	559.4 ± 31.9
K flat (SD), D	42.9 ± 1.5	42.9 ± 1.5	43.1 ± 1.5	42.5 ±1.1
K step (SD), D	43.4 ± 1.4	43.3 ± 1.6	43.5 ± 1.6	43.1 ± 1.1
ACD (mm)	2.81 ± 0.3	2.77 ± 0.3	2.78 ± 0.3	2.70 ± 0.3
AL(mm)	22.8 ± 0.7	22.9 ± 1.0	22.9 ± 1.0	23.2 ± 1.0
*RNFL thickness (μm)*				
G	103.9 ± 13.0	99.5 ± 13.2	98.1 ± 12.2	100.1 ± 9.54
T	76.4 ± 14	75.7 ± 19	71.4 ± 13.4	78.1 ± 15.8
Ts	136.6 ± 22.3	139.5 ± 26.6	133.1 ± 30.0	138.6 ± 15.8
Ti	144.1 ± 19.4	137.2 ± 26.1	136.2 ± 26.1	144.9 ± 21.6
N	79.5 ± 17.2	76.9 ± 19.1	74.8 ± 13.9	76.8 ± 15.8
Ns	113.3 ± 18.2	112.4 ± 20.1	107.5 ± 22.8	106.3 ± 17.6
Ni	107.3 ± 24.2	104.3 ± 26.2	105.5 ± 23.5	104.37 ± 23.9

IOP: intraocular pressure; CCT: central corneal thickness; K flat: corneal dioptric power in the flattest meridian; Ksteep: corneal dioptric power in the steepest meridian; ACD: anterior chamber depth; AL: axial length; RNFL: retinal nerve fiber layer; G: global, T: temporal, Ts: temporal superior, Ti: temporal inferior, N: nasal, Ns: nasal superior, Ni: nasal inferior

The mean IOP and the mean CCT of the patients with OSAS were lower than the control group. However, this difference was not statistically significant. No significant difference was found among all of the groups in terms of K flat, K steep, ACD, AL. In addition, we found that the differences between the mean values of RNFL thickness in all quadrants were similar in both OSAS and control groups (p=0.274).

The lowest mean RNFL thickness is the temporal quadrant in the severe OSAS group, (71.4 ± 13.4 μm). As mentioned in the previous studies,^12^ we found that gender has no influence on RNFL values (p=0.77).

We found no correlation between AHI, lowest oxygen saturation (LAST) or BMI and RNFL thickness (G,T,Ts,Ti,N,Ns,Ni), IOP or CCT when the OSAS group was divided according to its severity (Table-IV).

**Table-IV:** Correlation coefficients between AHI, LO_2_S or BMI and peripapillary RNFL thickness (G, T, Ts, Ti, N, Ns, Ni), IOP and CCT.

	G	T	Ts	Ti	N	Ns	Ni	IOP	CCT
AHI	0.268*	0.466*	0.210*	0.530*	0.270*	0.134*	0.924*	0.055*	0.671*
	-0.110**	-0.073**	-0.125**	-0.063**	-0.110**	-0.148**	-0.010**	0.190**	0.042**
LO_2_S	0.991*	0.142*	0.470*	0.369*	0.192*	0.856*	0.422*	0.446*	0.985*
	-0.001**	-0.146**	-0.072**	0.089**	0.130**	0.018**	0.080**	-0.076**	0.002**
BMI	0.904*	0.056*	0.789*	0.550*	0.097*	0.489*	0.594*	0.026*	0.278*
	-0.012**	0.189**	0.027**	-0.060**	-0.164**	-0.069**	-0.053**	0.219**	0.108**

IOP: intraocular pressure; CCT: central corneal thickness; RNFL: retinal nerve fiber layer; G: global; T: temporal; Ts: temporal superior; Ti: temporal inferior; N: nasal; Ns: nasal superior; Ni: nasal inferior; AHI: apnea–hypopnea index; LO_2_S: lowest oxygen saturation; BMI: body mass index

* p- value

** Correlation coefficient

## DISCUSSION

This study assessed changes in the cornea and retina, the two most sensitive hypoxia layers in the healthy control group with different severe OSAS patients. OSAS is a syndrome characterized by recurrent complete (apnea) or partial (hypopnea) episodes of upper airway obstruction during sleep, and frequent decrease in blood oxygen saturation (SpO_2_).^13^ These repetitive changes in SpO_2_ cause ischemia - reperfusion injury leading to oxidative stress and loss of function in the corneal endothelium. The most important function of endothelium is the fluid electrolyte pump, and this allows the corneal hydration to be maintained at 78%. Since endothelium ion passage is partly energy dependent, failure to obtain sufficient energy in an aerobic conditions results in corneal edema. In addition, the accumulation of hypocotyls between the ascending epithelial cells of the anaerobic glycolysis end, lactate is first passed through the endothelium to the stroma.

However, if the increase in the level of the ones in the form of lactate is more than the discharge, it gathers in the stroma by creating an osmotic load and corneal edema happens in there.^14^ The reason for 2% thinning in the cornea after edema has disappeared is the worsening and potential keratocyte death. It was documented that keratocyte loss may be observed due to chronic hypoxia in patient with extended contact lens wearer and this situation may cause reduction of corneal thickness.^15^ Another reason for corneal thinning is hypoxia-induced stromal acidosis. This is mainly caused by anaerobic glycolysis under the hypoxic conditions.^16^ Acute hypoxia can cause increase to central corneal thickness with swelling, while corneal swelling diminishes over time.^17^ The fact that a tissue using up excessive oxygen such as cornea is quite probable to be consistently hypoxic in OSAS cases, though our study found no significant difference in corneal thickness between the groups.^6^,^18^,^19^ However, in a study conducted by Koseoglu et al. with 195 participants, corneal thickness decreased in OSAS group compared to the control group. Besides, similarly, they found that CCT was adversely associated with Oxygen Desaturation Index (ODI) values, desaturation rates, and decidedly related with least O_2_ immersion values.^20^ In our study, no correlation between AHI, LO_2_S and BMI, CCT was found.

Maximum oxygen that retina requires occurs in darkness, particularly during night.^21^ The entrance of the vein to the outer part of the retina is caused by the choroid and from the inner part it is from the retinal vessels.

Choroid vessels are tissues with the fastest blood flow conversely, retinal vessels have slow blood flow and better oxygen uptake, and thus the interior of the retina is hypoxia sensitive.^12^,^22^ The frequent occurrence of hypoxemia at night may cause neuronal death and optic neuropathy by endangering optic nerve perfusion and oxygenation. Neuronal death may cause permanent loss of vision by loss of retinal ganglion cells (RGCs), reduction of the peripapillary retinal nerve fiber layer (RNFL) thickness, optic nerve head imperfections, and decreased visual field (VF) sensitivity.^23^,^24^ Peripapillary RNFL thickness measurement reflects neuronal axons, and would allow quantification of ganglion cell axonal loss.

We found that the differences between the mean values of RNFL thickness in all quadrants were similar in both OSAS and control groups. The lowest mean RNFL thickness is the temporal quadrant of the severe OSAS group, which is 71.4 ± 13.4 μm. Bayhan et al.^25^ reported that RNFL thickness was reduced in the severe OSAS group compared to nasal and upper quadrant controls. In the same study, nasal RNFL thickness measurement of the moderate OSAS group was also thinner than that of the controls. Sagiv et al.^26^ reported that the RNFL thickness of patients with advanced OSAS decreased in the upper and lower quadrants. However, the decrease is smaller than 5.2 μm, but this is a small difference and probably due to the variability of the spectral area OCT.^6^ Kargi et al.^27^ compared OSAS and RNFL measurements of the healthy group and found a significant decrease in the OSAS group. In Ozge et al. the mean RNFL thickness in both eyes in the OSAS group was significantly thinner than the RNFL thickness control group in the right eye.^19^ Previous studies have reported that individuals with OSAS did not have a decrease in RNFL thickness,^24^,^28^ while disease severity and RNFL thickness showed no correlation in others.^26^,^29^

We found no correlation between AHI, LAST or BMI and RNFL (G,T,Ts,Ti,N,Ns,Ni), IOP or CCT when the OSAS patients group was divided according to its severity. Lin et al.^30^ reported that RNFL in superior and nasal quadrants were negatively correlated with AHI in the regression analysis (r = −0.217, −0.173, respectively), minimum arteritic oxygen saturation were positively associated with average RNFL, superior RNFL, nasal RNFL (r = 0.260, 0.200, 0.156, respectively). Shiba et al.^31^ also reported a significant correlation of nasal RNFL thickness and AHI (r = −0.31) in Japanese subjects. A statistically significant relationship between AHI and RNFL thickness was also observed in a prospective cohort study.^32^ In Ozge et al., between AHI and mean RNFL thickness showed a median negative correlation (r= 0.411, p=0.001).^19^ However, Bayhan et al.^25^ and Casas et al.^33^ found no significant correlation between the AHI and RNFL thickness in both the OSAS group and the control group.

There was no significant difference in IOP between the four groups. IOP is the most important risk factor for the beginning and evolution of glaucoma. The higher IOP brings about a change in the optic nerve structure, followed by RNFL thinning.^34^ Other studies by Karakucuk et al.^35^ showed a positive correlation between IOP and AHI. In Goldblum et al., OSAS and patients with normal tension glaucoma did not have increased IOP after prolonged apnea compared to normal breathing periods.^36^

We suggest that the differences between the results obtained in different studies may be due to the demographic characteristics of the OSAS and the control group, the duration of OSAS, the vascular dysregulation present in patients with OSAS, the calibrations of measuring instruments and the measurement methods.

### Limitation of the study

The control group did not undergo an overnight sleep study because this examination is costly. However, the anamnesis and symptoms of OSAS were questioned in order to select the control group. Thus, the possibility of involving a patient with OSAS in the study was minimized.

## CONCLUSION

We found that peripapillary RNFL, CCT and IOP did not change with the severity of OSAS. We also found no correlation between apnea severity (AHI), lowest oxygen saturation (LAST) and BMI and RNFL, CCT and IOP. However, multicenter, case-control and longterm cohort studies are still needed to assess the changes of RNFL thickness, CCT and IOP in OSAS patients.

### Authors’ Contribution

**KT:** Design of the study, coordinated all work related to the study, and manuscript writing.

**MTE, EGB:** Did data collection.

**KT, MK, EGB:** Did review and final approval of manuscript.

**KT:** Did statistical analysis.
